# Supplementary material for: Measuring the strength of maternal, newborn and child health care implementation and its association with childhood mortality risk in three rural districts of Tanzania
Source: PLOS Glob Public Health. 2025 Nov 13;5(11):e0005346. doi: 10.1371/journal.pgph.0005346 (PMC12614556; doi:10.1371/journal.pgph.0005346)
Supplement: S1 Table — Model 1: Complete case analysis omitting covariates with missing data (n = 6,068). Model 2: Addresses missing data by imputing community-level median values of household SES and mothers’ years of schooling where data are missing (n = 8,999). Model 3: Addresses missing data by using multiple imputation with chained equations (n = 8,999). (DOCX) [file pgph.0005346.s001.docx]

**Supplemental File 1 (S1 Table)**

**Associations between child mortality risk and implementation strength scores (n=8999)**

|  | **Model 1** | | **Model 2** | | **Model 3** | |
| --- | --- | --- | --- | --- | --- | --- |
|  | HR | 95% CI | HR | 95% CI | HR | 95% CI |
| **IS scores** | | | | | | |
| IS score 1 | 0.62* | 0.40, 0.98 | 0.64* | 0.39, 0.95 | 0.59* | 0.37, 0.92 |
| IS score 2 | 0.54* | 0.32, 0.99 | 0.56* | 0.34, 0.96 | 0.50* | 0.27, 0.94 |
| IS score 3 | 0.48 | 0.16, 1.27 | 0.46 | 0.19, 1.32 | 0.49 | 0.20, 1.30 |
| **Child sex** | | | | | | |
| Female | - | - | - | - | - | - |
| Male | 1.25* | 1.05, 1.48 | 1.25* | 1.05, 1.49 | 1.25* | 1.05, 1.48 |
| **Birth order** | | | | | | |
| No. children (cont.) | 1.00 | 0.88, 1.13 | 1.00 | 0.88, 1.13 | 1.00 | 0.88, 1.13 |
| **Previous birth interval** | | | | | | |
| Months (cont.) | 1.00 | 0.99, 1.00 | 1.00 | 0.99, 1.00 | 1.00 | 0.99, 1.01 |
| **Subsequent birth interval** | | | | | | |
| Months (cont.) | 1.01*** | 1.01, 1.02 | 1.01*** | 1.00, 1.02 | 1.01*** | 1.00, 1.02 |
| **Mother age at birth** | | | | | | |
| Years (cont). | 1.00 | 0.99, 1.01 | 1.01 | 0.99, 1.01 | 1.01 | 0.99, 1.02 |
| **Mother marital status at birth** | | | | | | |
| Married/in union | - | - | - | - |  |  |
| Single | 1.43** | 1.19, 1.71 | 1.43** | 1.19, 1.72 | 1.43** | 1.19, 1.71 |
| **Mother number years of schooling** | | | | | | |
| Year of schooling (cont). | - | - | 0.97 | 0.96, 1.01 | 0.96 | 0.94, 1.01 |
| **Household SES at birth (quintile ranking)** | | | | | | |
| Fifth | - | - | - | - | - | - |
| Fourth | - | - | 0.87 | 0.62, 1.22 | 0.79^ꞎ^ | 0.60, 1.03 |
| Third | - | - | 1.06 | 0.87, 1.69 | 0.99 | 0.75, 1.29 |
| Second | - | - | 1.06 | 0.75, 1.51 | 0.95 | 0.71, 1.26 |
| First | - | - | 0.97 | 0.67, 1.41 | 0.93 | 0.70, 1.25 |
| **Distance to nearest hospital** | | | | | | |
| Kilometers (cont.) | 1.04^ꞎ^ | 1.00, 1.07 | 1.14^ꞎ^ | 0.99, 1.32 | 1.15^*^ | 1.00, 1.34 |
| **HDSS zone** | | | | | | |
| Ifakara Expansion | - | - | - | - | - | - |
| Ifakara Rural | 0.82 | 0.58, 1.18 | 0.85 | 0.58, 1.23 | 0.83 | 0.58, 1.20 |
| Ifakara Urban | 1.39 | 0.92, 2.14 | 1.35 | 0.87, 2.06 | 1.35 | 0.87, 2.10 |
| Rufiji Rural | 1.05 | 0.65, 1.69 | 1.10 | 0.69, 1.79 | 1.08 | 0.68, 1.74 |
| Rufiji Urban | 0.90 | 0.51, 1.63 | 0.93 | 0.54, 1.68 | 0.93 | 0.53, 1.67 |
| **Community Health Worker Deployed to Community** | | | | | | |
| Yes | 0.94 | 0.74, 1.17 | 0.94 | 0.77, 1.15 | 0.95 | 0.77, 1.16 |

^ꞎ = p-values < 0.1, * = p-values < 0.05, 0.** = p-values <0.01, *** = p-values <0.001^
