# Supplementary material for: Measuring the strength of maternal, newborn and child health care implementation and its association with childhood mortality risk in three rural districts of Tanzania
Source: PLOS Glob Public Health. 2025 Nov 13;5(11):e0005346. doi: 10.1371/journal.pgph.0005346 (PMC12614556; doi:10.1371/journal.pgph.0005346)
Supplement: S2 Table — (DOCX) [file pgph.0005346.s002.docx]

**Supplementary File 2 (S2 Table)**

|  | Ifakara Rural | Ifakara Urban | Ifakara Expansion | Rufiji Rural | Rufiji Urban |
| --- | --- | --- | --- | --- | --- |
| General (staffing, management, infrastructure) | 0.71 (0.25, 0.99) | 0.71 (0.61, 0.99) | 0.75 (0.57, 0.99) | 0.46 (0.33, 0.72) | 0.72 (0.58, 0.99) |
| Family planning | 0.28 (0.06, 0.50) | 0.18 (0.06, 0.43) | 0.23 (0.08, 0.49) | 0.14 (0.00, 0.44) | 0.21 (0.08, 0.43) |
| Antenatal care | 0.29 (0.16, 0.49) | 0.26 (0.12, 0.47) | 0.23 (0.10, 0.48) | 0.13 (0.05, 0.64) | 0.23 (0.14, 0.70) |
| Intrapartum care | 0.25 (0.08, 0.49) | 0.39 (0.15, 0.54) | 0.34 (0.15, 0.57) | 0.33 (0.14, 0.56) | 0.45 (0.20, 0.75) |
| Postnatal care | 0.12 (0.00, 0.30) | 0.15 (0.06. 0.28) | 0.17 (0.08, 0.28) | 0.16 (0.00, 0.38) | 0.26 (0.09, 0.37) |
| Preventive childhood services | 0.82 (0.27, 0.99) | 0.72 (0.27, 0.99) | 0.68 (0.22, 0.90) | 0.53 (0.28, 0.83) | 0.63 (0.27, 0.90) |
| Sick childcare | 0.30 (0.13, 0.54) | 0.36 (0.17, 0.69) | 0.33 (0.15, 0.48) | 0.21 (0.13, 0.45) | 0.30 (0.19, 0.46) |
