# Supplementary material for: Measuring the strength of maternal, newborn and child health care implementation and its association with childhood mortality risk in three rural districts of Tanzania
Source: PLOS Glob Public Health. 2025 Nov 13;5(11):e0005346. doi: 10.1371/journal.pgph.0005346 (PMC12614556; doi:10.1371/journal.pgph.0005346)
Supplement: S3 Table — (DOCX) [file pgph.0005346.s003.docx]

**Supplemental File 5 (S5 Table)**

|  | **Newborn mortality**  **(<1 month)** | | **Infant mortality (<12 months)** | | **Child mortality**  **(<60 months)** | |
| --- | --- | --- | --- | --- | --- | --- |
|  | HR | 95% CI | HR | 95% CI | HR | 95% CI |
| **IS scores** | | | | | | |
| IS score 1 (ANC, PNC and preventive child care) | 0.56 | 0.27, 1.19 | 0.65 | 0.37, 1.14 | 0.59* | 0.37, 0.92 |
| IS score 2 (sick child care) | 0.45 | 0.16, 1.22 | 0.51^ꞎ^ | 0.24, 1.09 | 0.53* | 0.30, 0.97 |
| IS score 3 (intrapartum care) | 0.59 | 0.12, 2.84 | 0.44 | 0.14, 1.44 | 0.51 | 0.20, 1.30 |
| **Child sex** | | | | | | |
| Female | - | - | - | - | - | - |
| Male | 1.10 | 0.85, 1.43 | 1.13 | 0.92, 1.38 | 1.25* | 1.05, 1.48 |
| **Birth order** | | | | | | |
| No. children (cont.) | 1.20* | 1.01, 1.44 | 1.13 | 0.98, 1.30 | 1.00 | 0.88, 1.13 |
| **Previous birth interval** | | | | | | |
| Months (cont.) | 1.00 | 0.99, 1.00 | 1.00 | 0.99, 1.00 | 1.00 | 0.99, 1.01 |
| **Subsequent birth interval** | | | | | | |
| Months (cont.) | 1.01** | 1.00, 1.02 | 1.01*** | 1.00, 1.02 | 1.01*** | 1.00, 1.02 |
| **Mother age at birth** | | | | | | |
| Years (cont). | 1.00 | 0.98, 1.01 | 1.00 | 0.98, 1.01 | 1.01 | 0.99, 1.02 |
| **Mother marital status at birth** | | | | | | |
| Married/in union | - | - | - | - |  |  |
| Single | 1.60** | 1.22, 2.11 | 1.42** | 1.15, 1.75 | 1.43** | 1.19, 1.71 |
| **Mother number years of schooling** | | | | | | |
| Year of schooling (cont). | 1.01 | 0.97, 1.06 | 0.98 | 0.94, 1.01 | 0.96 | 0.94, 1.01 |
| **Household SES at birth (quintile ranking)** | | | | | | |
| Fifth | - | - | - | - | - | - |
| Fourth | 1.01 | 0.67, 1.54 | 0.86 | 0.62, 1.19 | 0.79^ꞎ^ | 0.60, 1.03 |
| Third | 1.19 | 0.77, 1.81 | 1.18 | 0.86, 1.61 | 0.99 | 0.75, 1.29 |
| Second | 0.98 | 0.63, 1.55 | 1.00 | 0.72, 1.40 | 0.95 | 0.71, 1.26 |
| First | 1.03 | 0.48, 2.24 | 1.03 | 0.73, 1.46 | 0.93 | 0.70, 1.25 |
| **Distance to nearest hospital** | | | | | | |
| Kilometers (cont.) | 1.07 | 0.765, 1.78 | 1.06 | 0.88, 1.6 | 1.15^*^ | 1.00, 1.34 |
| **HDSS zone** | | | | | | |
| Ifakara Expansion | - | - | - | - | - | - |
| Ifakara Rural | 0.68 | 0.38, 1.25 | 0.68^ꞎ^ | 0.44, 1.06 | 0.83 | 0.58, 1.20 |
| Ifakara Urban | 1.12 | 0.53, 2.34 | 1.40 | 0.81, 2.44 | 1.35 | 0.87, 2.10 |
| Rufiji Rural | 0.84 | 0.39, 1.83 | 0.77 | 0.43, 1.40 | 1.08 | 0.68, 1.74 |
| Rufiji Urban | 0.77 | 0.31, 1.94 | 0.67 | 0.33, 1.38 | 0.93 | 0.53, 1.67 |
| **Community Health Worker Deployed to Community** | | | | | | |
| Yes | 0.97 | 0.69, 1.37 | 0.91 | 0.71, 1.18 | 0.95 | 0.77, 1.16 |

ꞎ = p-values < 0.1, * = p-values < 0.05, 0.** = p-values <0.01, *** = p-values <0.001
