# Supplementary figures and images for: Measuring the strength of maternal, newborn and child health care implementation and its association with childhood mortality risk in three rural districts of Tanzania
Source: PLOS Glob Public Health. 2025 Nov 13;5(11):e0005346. doi: 10.1371/journal.pgph.0005346 (PMC12614556; doi:10.1371/journal.pgph.0005346)

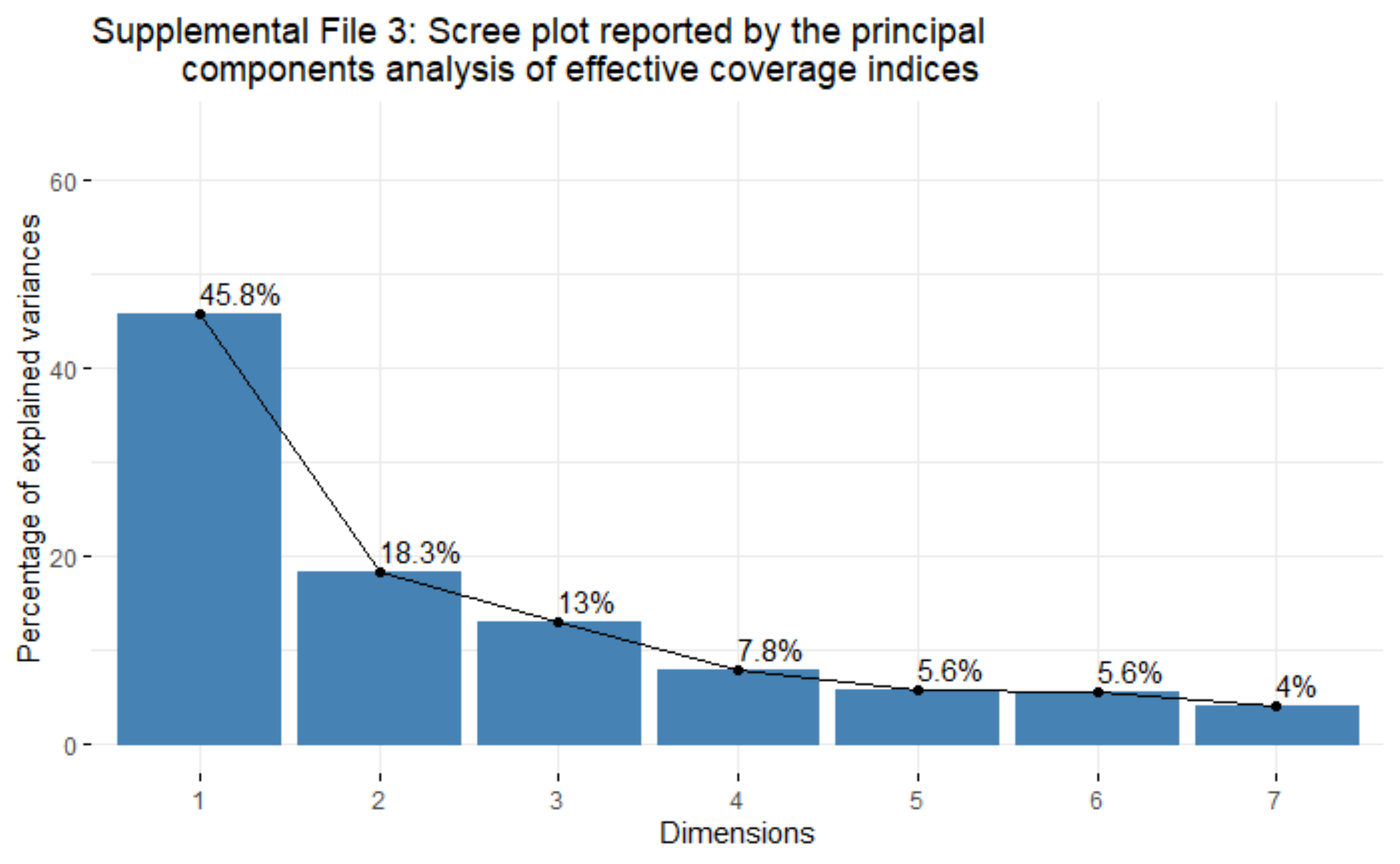

Supplement: S1 Fig — (TIF) [file pgph.0005346.s004.tif]
