# Supplementary material for: Measuring the strength of maternal, newborn and child health care implementation and its association with childhood mortality risk in three rural districts of Tanzania
Source: PLOS Glob Public Health. 2025 Nov 13;5(11):e0005346. doi: 10.1371/journal.pgph.0005346 (PMC12614556; doi:10.1371/journal.pgph.0005346)
Supplement: S2 Fig — (PDF) [file pgph.0005346.s005.pdf]

Supplemental File 4a: Distribution of implementation strength scale 1's median values across geographic zones within local health systems in the study area

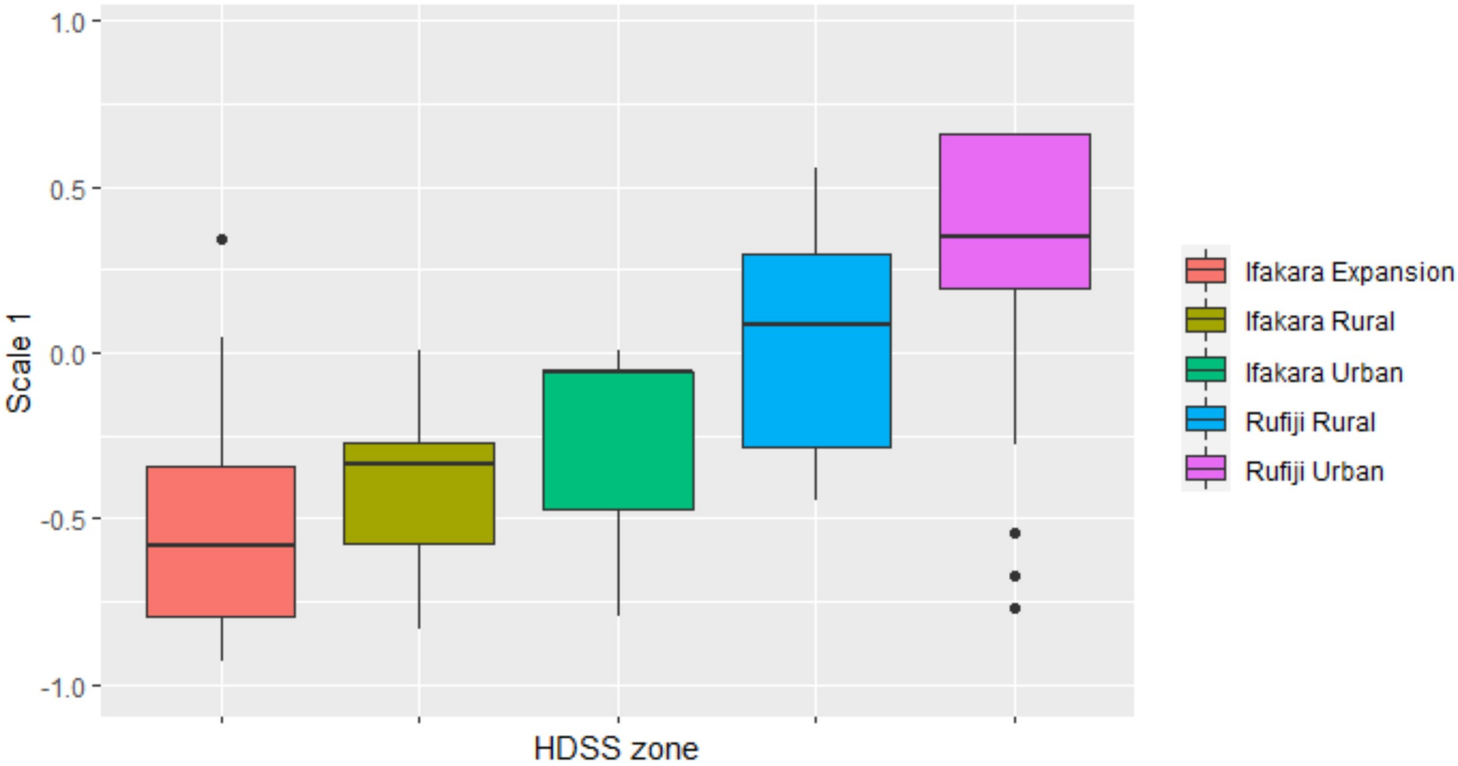

Scale 1 is of availability and readiness to provide preventive services (mostly child related)

Supplemental File 4b: Distribution of implementation strength scale 2's median values across geographic zones within the study area

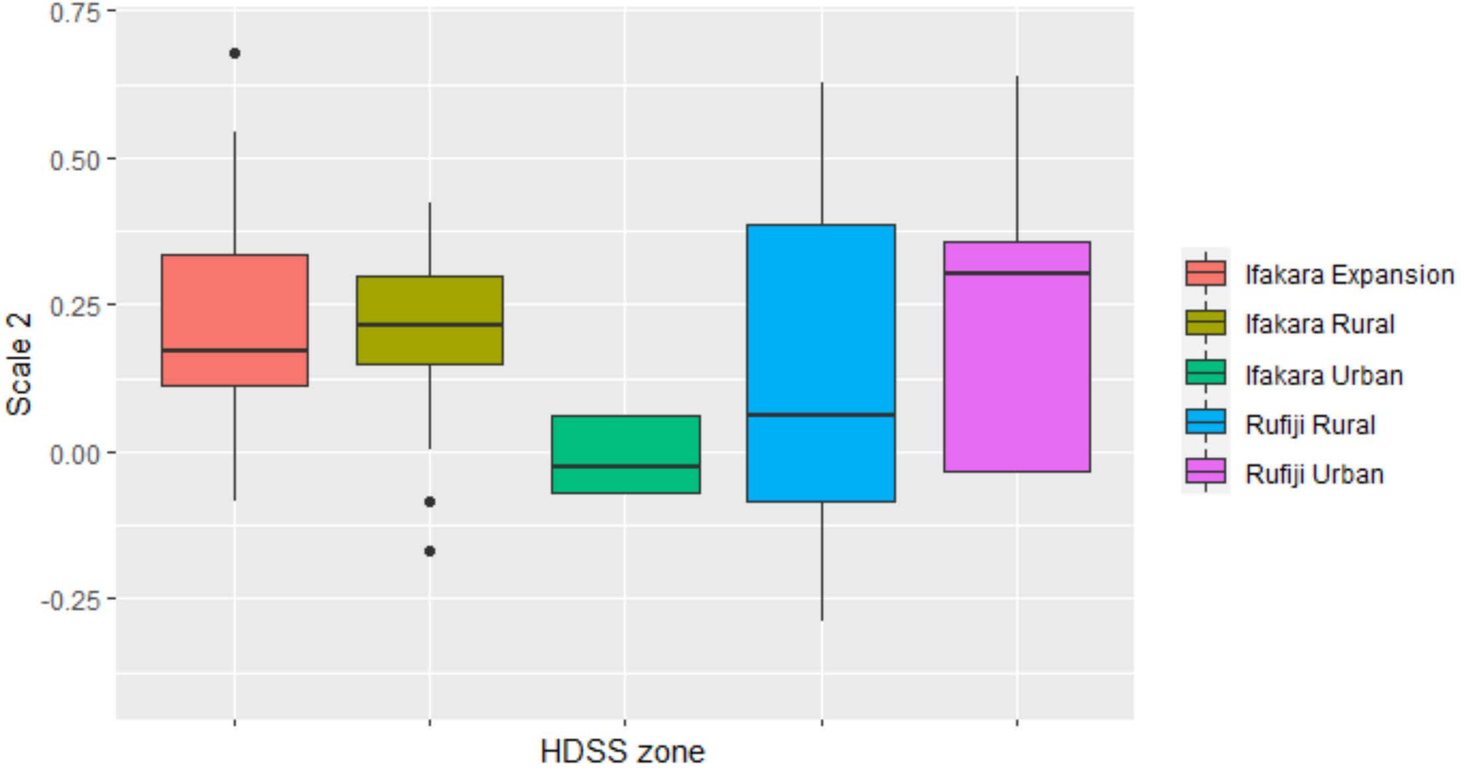

Scale 2 reflects availability and readiness to provide sick child care and family planning services

Supplemental File 4c: Distribution of implementation strength scale 3's median values across geographic zones within the study area

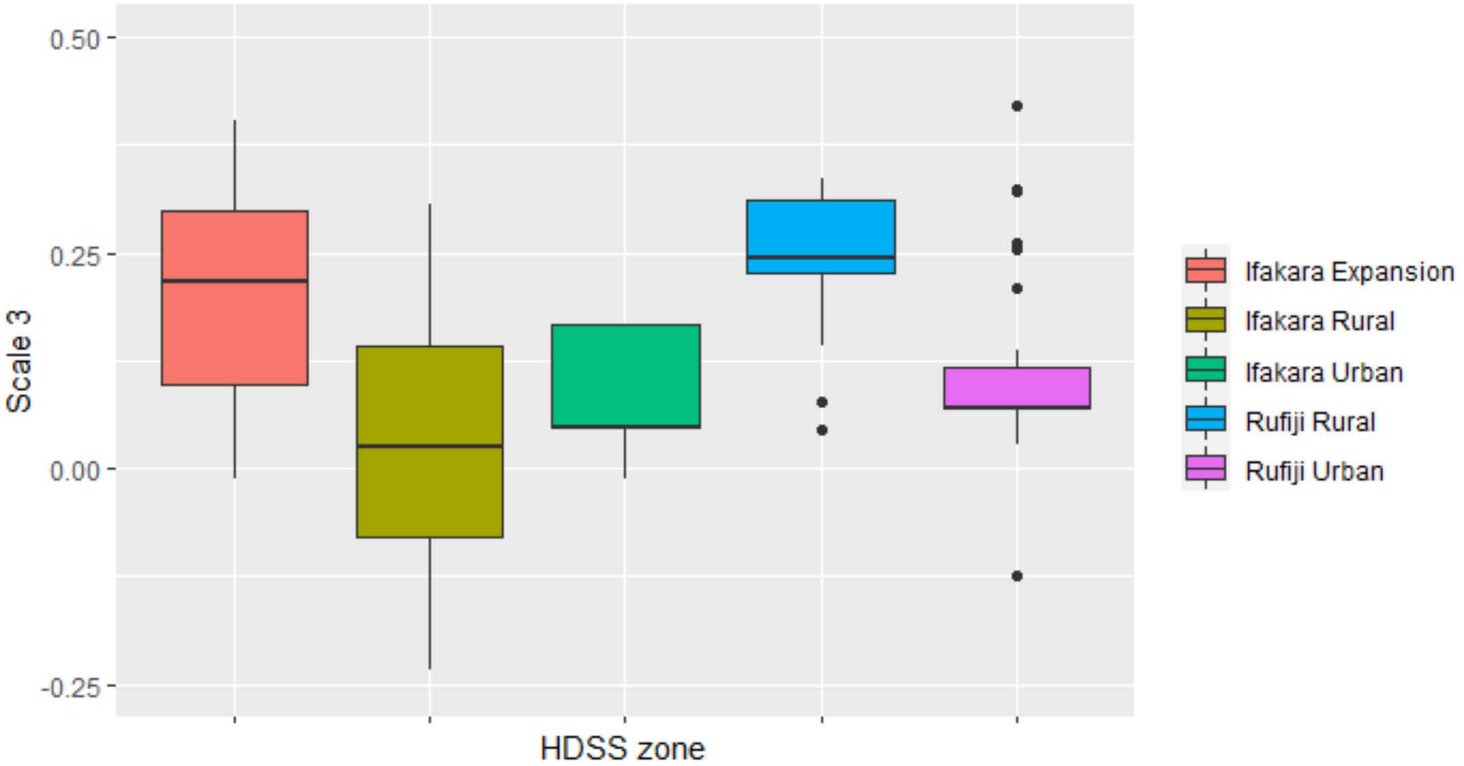

Scale 3 reflects availability and readiness to provide intrapartum care
