# Supplementary material for: Measuring the strength of maternal, newborn and child health care implementation and its association with childhood mortality risk in three rural districts of Tanzania
Source: PLOS Glob Public Health. 2025 Nov 13;5(11):e0005346. doi: 10.1371/journal.pgph.0005346 (PMC12614556; doi:10.1371/journal.pgph.0005346)
Supplement: Attachment — Submitted filename: Response to Reviewers (9 28).odt [file pgph.0005346.s007.odt]

Response to Reviewers
Below, we provide a table that outlines how we have responded to each point raised by Reviewer #1.  The page numbers discussed in the table refer to the ‘tracked change’ version of the revised manuscript that we submit herewith.  
After the table, we provide more information on revisions that were made to the references and reference section of the manuscript.
No.
Comment
Response
Abstract
1
The abstract directly transitions into the study design without providing a brief context of rationale (1-2 sentences) for why assessing the link between the implementation strength of MNCH services and child survival is important.
This is addressed on page 2, lines 38-42 of the revised manuscript.
2
Technical details like “gradients of the collective implementation strength exerted by groups of facilities on villages they served using Bayesian mixed effects models” are excessive for an abstract and could be simplified into plain language.
This is addressed on page 2, lines 47-51 of the revised manuscript.
3
The results are not presented with standard scientific rigor. The phrase “0.59 times lower” and “0.50 times lower” should be expressed as hazard ratios (HR) or relative risks (RR) with confidence intervals, e.g. “HR = 0.59, 95% CI = X-Y”.
This is addressed on page 2-3, lines 57-61 of the revised manuscript.
4
The abstract reads more like a methods-heavy summary than a balanced abstract. Consider rebalancing the content to devote ~25% to background, 35-40% to methods and results and 25-30% to conclusion/implications.
After revising the abstract, it is rebalanced to devote 25% to background, 25% to conclusions/ implications and 50% to methods. We hope this is acceptable.
Introduction
1
The introduction effectively highlights methodological challenges (e.g., cross-sectional limitations, ecological bias), but it does not clearly mention existing controversies or debates in the literature about how best to measure "implementation strength." This could be strengthened by citing opposing viewpoints or gaps in consensus.
This is addressed on page 4, lines 84-90 of the revised manuscript.
2
The flow from global child mortality trends (lines 61–64) to the methodological challenges (lines 94–134) could be improved by inserting a clear research gap statement early on.
This is addressed on page 4, lines 82-83 of the revised manuscript.
3
The term "implementation strength" is defined multiple times (lines 73–76 and 94–103). It should be defined once early, with subsequent mentions focusing on challenges in measuring it.
Thanks for the comment. Respectfully, we do feel that the term was defined once early (In the original manuscript, the term was defined once, early in lines 73-76, which is the first sentence of the second paragraph of the introduction (90-93 in the revised manuscript) 

The subsequent text describes how the concept of implementation strength has been applied over time (lines 76-93 in original manuscript, 93-110 in revised manuscript) and challenges that researchers have faced when trying to measure it (94-134 of original, 111-151 of the revised manuscript).

We are grateful for the comment but feel that the manuscript does not require any revision to address it.
4
This section is too detailed and partially belongs in the Methods (e.g., SARA timeline, number of children). The journal guidelines suggest ending the introduction with a concise aim and statement of achievement.
Our revisions to the section “Implementation strength: Measurement and association with child health gains” reduces the length of this section and its level of detail.  See page 5, lines 112-151 of the revised manuscript.
5
Technical terms like "dose strength," "signal function framework," and "Balanced ScoreCard" should be briefly defined or simplified for readers outside global health.
Thanks for the comment. Respectfully, we believe that the term dose was defined in the original manuscript (line 75, line 92 of revised manuscript). Similarly, the signal function framework was defined as a set of performance indicators that are used to evaluate maternal survival programs in the absence of sufficient mortality data (lines 77-78 of original manuscript, 94-96 of revised manuscript). Also, the Balanced Scorecard is defined as dashboard of performance indicators that are used to detect variation and change in PHC implementation strength (on lines 90-91 of original manuscript, 106-108 of revised manuscript). 

We provided references for readers to turn to if they are still unclear on the meaning of these terms. While we agree that it is important to consider the readers’ perspective, we also believe that the readers of PLoS Global Public Health will be in the global health field.
6
The Introduction does not adequately highlight Tanzania's child mortality rates or trends, e.g., under-five mortality rates, neonatal mortality, or progress towards Millennium Development Goals (MDGs).
This is addressed on page 7 of the revised manuscript (lines 154-161).
7
While global trends are mentioned, there is no concrete data on the current child mortality context in Tanzania (e.g., comparing rural vs. urban or trends over the past decades).
This is addressed on page 7 of the revised manuscript (lines 154-161).
8
There is no quantitative link or historical trend data showing how prior PHC or IMCI strategies affected child survival in Tanzania.
This is addressed on page 9 of the revised manuscript (lines 199-201).
Methodology
1
While household and facility sampling procedures are important, details like “data collectors approached them in their homes” (line 194) or “data collectors used GPS tracking devices” (line 199) can be condensed.
The revisions made between page 9 and 11, lines 203-243 of the revised manuscript address this comment.
2
Retain only essential sampling criteria (eligibility, random selection) and move operational details to Supplementary Materials.
The revisions made between page 9 and 11, lines 203-243 of the revised manuscript address this comment.
3
Line 161–172: This subsection reads more like historical background than a methodological description. While it gives context, much of it (e.g., 1970s PHC expansion, free access policy) could be condensed to a few sentences.
The revisions made on page 8, lines 187-201 of the revised manuscript address this comment.
4
The decline in child mortality (line 173–174) is relevant but might be better placed in the Introduction.
This is addressed on page 7 of the revised manuscript (lines 154-161).
5
Line 183–184: Ensure that Figure 1 is clearly labeled and explained in the manuscript.
This is addressed on page 9, lines 210-212 of the revised manuscript.
6
Line 165–172: A well-sequenced policy timeline is presented. The inclusion of IMCI and skilled obstetric care is important, though the text could better clarify whether all services were uniformly implemented nationwide or varied by region.
This is an excellent point; however, after some research we were unable to obtain information that would allow to explain concisely whether IMCI implementation varied by region during the period before the Connect trial. Since Reviewer #1 has advised us to reduce the length and level of detail of this section (see third comment to the Methodology section), we respectfully ask that we be permitted not to provide these details.
7
Line 187–194: Household survey methods are generally well described. The selection method within villages ("randomly selected") is unclear whether it was stratified or simple random sampling.
This is addressed on page 10, lines 219-222 of the revised manuscript.
8
The HDSS structure provides high-quality longitudinal data; however, reliance on household informants for demographic events (especially migration) could introduce recall or reporting bias. Could the authors clarify how this potential bias was minimized or accounted for during data collection and analysis?
This is addressed on page 28, lines 593-597 (in the discussion of study limitations).
9
Limiting the analysis to PHC facilities is reasonable given the focus on implementation strength, and the exclusion of hospitals is logically consistent with this aim. However, could the authors explicitly acknowledge how this exclusion may constrain the generalizability of their findings, particularly for settings where hospital-level care significantly impacts child outcomes?
This is addressed on pages 28-29, lines 597-600 (in the discussion of study limitations).
10
The use of Cronbach’s alpha (≥0.8) to assess internal consistency of indicators within each domain is appropriate. However, Cronbach’s alpha alone does not provide evidence of content or construct validity. Consider acknowledging these limitations or complementing alpha with expert review, factor analysis, or triangulation with external data.
This is addressed on pages 27-28, lines 573-575 (in the discussion of study limitations).
11
Line 255–258 - Use of cosine² values is uncommon in public health literature and may not be intuitive to all readers. A short clarification in the results could make it more accessible (e.g., “cosine² values indicate how well each domain contributes to a principal component”).
This is addressed on page 13, line 289 of the revised manuscript.
Results
1
The transition between descriptive results and analytical outcomes (e.g., from PCA to hazard ratios) could be made smoother, with brief interpretative statements that connect how PCA-derived scales feed into the mortality models.
This is addressed on page 21, lines 437-442, in the revised manuscript.							
2
Table 3: The cohort data (n = 8,999, with 526 deaths) is well described, but the high loss to follow-up due to outmigration (43%) should be highlighted in the results as it could affect mortality estimates (even if adjusted analytically).
This is addressed on page 19, lines 429-432, in the revised manuscript.									
3
Lines 421–425: The additional analysis for neonatal and infant mortality (not statistically significant) is valid but mentioned too briefly. A supplemental table reference (S5 Appendix) is good, but a one-line mention of sample size/power issues would contextualize the non-significance.
This is addressed on page 23, lines 476-480 of the revised manuscript (discussion section).  

While the reviewer makes an excellent point, we feel that the discussion section is the more appropriate place to reflect on this issue. To avoid repetition, we respectfully wish to raise this important point in the discussion section only.
4
Time scale (age vs time since birth) and left-truncation/right-censoring handling details are missed.
This is addressed on pages 14-15, lines 328-338 of the revised manuscript.
5
The analysis does not assess effect modification (interaction) to determine whether associations between implementation strength and child mortality differ by socio-economic status, distance to hospital, or geographic zone. Including such checks could reveal important subgroup variations and strengthen interpretation of results.
While we agree that this is relevant, we feel that the analysis is already very complex and that these results might complicate the interpretation of findings more so that they would enhance the meaningfulness of the analysis.  Therefore, we respectfully request that we be permitted not to include this analysis.

However, if the Editor and reviewer feel strongly about including this analysis, we are open to doing it in a subsequent revision.
Discussion
1
The explanation for the lack of association with neonatal and infant mortality is attributed mainly to sample size (lines 432–434). While this is plausible, other reasons (e.g., quality of intrapartum care, referral system issues) could be briefly discussed for completeness.
This is addressed on page 23, line 478 of the revised manuscript.
2
The authors suggest that inclusion of hospitals might have revealed stronger effects, but they do not explore other structural or process factors (e.g., quality of emergency obstetric care) that might explain the null results.
This is addressed on page 24, line 493-497 of the revised manuscript.
3
They acknowledge PCA limitations (lines 518–522), but the rationale for using a hybrid approach (weighted-additive + PCA) could be better justified in the discussion rather than only in the methods.
This is addressed on page 28, lines 575-581 of the revised manuscript.
4
The authors note that the data are over 10 years old (lines 542–544) but argue that the interventions are still relevant. This argument is valid but could be strengthened by referencing current trends in PHC in Tanzania or citing updated DHS/MNCH program data.
This is addressed on page 29, lines 607-617 of the revised manuscript.

We would also like to point out some revisions that were made to the references.  
Reference number 1 was incorrect in the original version of the manuscript that was submitted. This has been corrected. See lines 32, 670-672 of the revised manuscript.
During revisions, we added reference #13.  See page 33, lines 714-716.
During revisions, we added references #48-49. See page 36, lines 825-830.
During revisions, we removed reference #50 (The SARA Project Management Group, 2013).
During revisions, we added reference #63. See page 36, lines 875-876.
During revisions, we added reference #87. See page 36, lines 943-948.
