# Supplementary material for: Measuring the strength of maternal, newborn and child health care implementation and its association with childhood mortality risk in three rural districts of Tanzania
Source: PLOS Glob Public Health. 2025 Nov 13;5(11):e0005346. doi: 10.1371/journal.pgph.0005346 (PMC12614556; doi:10.1371/journal.pgph.0005346)
Supplement: Peer review history

# Peer review: Decision Letter 0

**DOI:** 10.1371/journal.pgph.0005346.r001

**Authors:** Shiyam Sunder

5 Aug 2025

PGPH-D-25-01695

Measuring the strength of maternal, newborn and child health care implementation and its association with childhood mortality risk in three rural districts of Tanzania.

PLOS Global Public Health

Dear Dr. Baynes,

Thank you for submitting your manuscript to PLOS Global Public Health. After careful consideration, we feel that it has merit but does not fully meet PLOS Global Public Health’s publication criteria as it currently stands. Therefore, we invite you to submit a revised version of the manuscript that addresses the points raised during the review process.

Please submit your revised manuscript by Sep 04 2025 11:59PM. If you will need more time than this to complete your revisions, please reply to this message or contact the journal office at globalpubhealth@plos.org. When you're ready to submit your revision, log on to https://www.editorialmanager.com/pgph/ and select the 'Submissions Needing Revision' folder to locate your manuscript file.

Please include the following items when submitting your revised manuscript:

- A rebuttal letter that responds to each point raised by the editor and reviewer(s). You should upload this letter as a separate file labeled 'Response to Reviewers'.
- A marked-up copy of your manuscript that highlights changes made to the original version. You should upload this as a separate file labeled 'Revised Manuscript with Track Changes'.
- An unmarked version of your revised paper without tracked changes. You should upload this as a separate file labeled 'Manuscript'.

Guidelines for resubmitting your figure files are available below the reviewer comments at the end of this letter.

We look forward to receiving your revised manuscript.

Kind regards,

Shiyam Sunder, MBBS, MSc epidemiology, PhD

Academic Editor

PLOS Global Public Health

Journal Requirements:

1. Please include the following request in the decision letter, and ping me with follow-up. “Please include a complete copy of PLOS’ questionnaire on inclusivity in global research in your revised manuscript. Our policy for research in this area aims to improve transparency in the reporting of research performed outside of researchers’ own country or community. The policy applies to researchers who have travelled to a different country to conduct research, research with Indigenous populations or their lands, and research on cultural artefacts. The questionnaire can also be requested at the journal’s discretion for any other submissions, even if these conditions are not met. Please find more information on the policy and a link to download a blank copy of the questionnaire here: https://journals.plos.org/globalpublichealth/s/best-practices-in-research-reporting. Please upload a completed version of your questionnaire as Supporting Information when you resubmit your manuscript.

If the reviewer comments include a recommendation to cite specific previously published works, please review and evaluate these publications to determine whether they are relevant and should be cited. There is no requirement to cite these works unless the editor has indicated otherwise. 

Please review your reference list to ensure that it is complete and correct. If you have cited papers that have been retracted, please include the rationale for doing so in the manuscript text, or remove these references and replace them with relevant current references. Any changes to the reference list should be mentioned in the rebuttal letter that accompanies your revised manuscript. If you need to cite a retracted article, indicate the article’s retracted status in the References list and also include a citation and full reference for the retraction notice.

Additional Editor Comments (if provided):

[Note: HTML markup is below. Please do not edit.]

Reviewers' comments:

Reviewer's Responses to Questions

**Comments to the Author**

1. Does this manuscript meet PLOS Global Public Health’s publication criteria?

Reviewer #1: Yes

Reviewer #2: No

**********

2. Has the statistical analysis been performed appropriately and rigorously?

Reviewer #1: Yes

Reviewer #2: No

**********

3. Have the authors made all data underlying the findings in their manuscript fully available (please refer to the Data Availability Statement at the start of the manuscript PDF file)?

The PLOS Data policy

Reviewer #1: Yes

Reviewer #2: Yes

**********

4. Is the manuscript presented in an intelligible fashion and written in standard English?

Reviewer #1: Yes

Reviewer #2: Yes

**********

Reviewer #1: The study is well-designed and addresses an important gap by linking the implementation strength of MNCH services with child survival, offering valuable insights for health system performance in Tanzania. With some polishing in the presentation of methods, results, and interpretation, the manuscript can significantly contribute to understanding how strengthening PHC delivery can further reduce child mortality and inform policy improvements in Tanzania.

Abstract

1. The abstract directly transitions into the study design without providing a brief context or rationale (1–2 sentences) for why assessing the link between the implementation strength of MNCH services and child survival is important.

2. Technical details like “gradients of the collective implementation strength exerted by groups of facilities on villages they served using Bayesian mixed effects models” are excessive for an abstract and could be simplified into plain language.

3. The results are not presented with standard scientific rigor. The phrases "0.59 times lower" and "0.50 times lower" should be expressed as hazard ratios (HR) or relative risks (RR) with confidence intervals, e.g., "HR = 0.59, 95% CI = X-Y."

4. The abstract reads more like a methods-heavy summary than a balanced abstract. Consider rebalancing the content to devote ~25% to background, 35–40% to methods and results, and 25–30% to conclusions/implications.

Introduction

1. The introduction effectively highlights methodological challenges (e.g., cross-sectional limitations, ecological bias), but it does not clearly mention existing controversies or debates in the literature about how best to measure "implementation strength." This could be strengthened by citing opposing viewpoints or gaps in consensus.

2. The flow from global child mortality trends (lines 61–64) to the methodological challenges (lines 94–134) could be improved by inserting a clear research gap statement early on.

3. The term "implementation strength" is defined multiple times (lines 73–76 and 94–103). It should be defined once early, with subsequent mentions focusing on challenges in measuring it.

4. This section is too detailed and partially belongs in the Methods (e.g., SARA timeline, number of children). The journal guidelines suggest ending the introduction with a concise aim and statement of achievement.

5. Technical terms like "dose strength," "signal function framework," and "Balanced ScoreCard" should be briefly defined or simplified for readers outside global health.

6. The Introduction does not adequately highlight Tanzania's child mortality rates or trends, e.g., under-five mortality rates, neonatal mortality, or progress towards Millennium Development Goals (MDGs).

7. While global trends are mentioned, there is no concrete data on the current child mortality context in Tanzania (e.g., comparing rural vs. urban or trends over the past decades).

8. There is no quantitative link or historical trend data showing how prior PHC or IMCI strategies affected child survival in Tanzania.

Methodology

• While household and facility sampling procedures are important, details like “data collectors approached them in their homes” (line 194) or “data collectors used GPS tracking devices” (line 199) can be condensed.

• Retain only essential sampling criteria (eligibility, random selection) and move operational details to Supplementary Materials.

• (161–172 )This subsection reads more like historical background than a methodological description. While it gives context, much of it (e.g., 1970s PHC expansion, free access policy) could be condensed to a few sentences.

• The decline in child mortality (line 173–174) is relevant but might be better placed in the Introduction.

• Line 183–184: Ensure that Figure 1 is clearly labeled and explained in the manuscript.

• (165–172) A well-sequenced policy timeline is presented. The inclusion of IMCI and skilled obstetric care is important, though the text could better clarify whether all services were uniformly implemented nationwide or varied by region.

• 187–194: Household survey methods are generally well described. The selection method within villages ("randomly selected") is unclear whether it was stratified or simple random sampling.

• The HDSS structure provides high-quality longitudinal data; however, reliance on household informants for demographic events (especially migration) could introduce recall or reporting bias. Could the authors clarify how this potential bias was minimized or accounted for during data collection and analysis?

Here’s an improved version of your comment:

• Limiting the analysis to PHC facilities is reasonable given the focus on implementation strength, and the exclusion of hospitals is logically consistent with this aim. However, could the authors explicitly acknowledge how this exclusion may constrain the generalizability of their findings, particularly for settings where hospital-level care significantly impacts child outcomes?

• (225–227 )The use of Cronbach’s alpha (≥0.8) to assess internal consistency of indicators within each domain is appropriate. However, Cronbach’s alpha alone does not provide evidence of content or construct validity. Consider acknowledging these limitations or complementing alpha with expert review, factor analysis, or triangulation with external data.

• (255–258) Use of cosine² values is uncommon in public health literature and may not be intuitive to all readers. A short clarification in the results could make it more accessible (e.g., “cosine² values indicate how well each domain contributes to a principal component”).

Results (Lines 331–426)

1. The transition between descriptive results and analytical outcomes (e.g., from PCA to hazard ratios) could be made smoother, with brief interpretative statements that connect how PCA-derived scales feed into the mortality models.

2. Table 3: The cohort data (n = 8,999, with 526 deaths) is well described, but the high loss to follow-up due to outmigration (43%) should be highlighted in the results as it could affect mortality estimates (even if adjusted analytically).

3. Lines 421–425: The additional analysis for neonatal and infant mortality (not statistically significant) is valid but mentioned too briefly. A supplemental table reference (S5 Appendix) is good, but a one-line mention of sample size/power issues would contextualize the non-significance.

4. Time scale (age vs time since birth) and left-truncation/right-censoring handling details are missed.

5. The analysis does not assess effect modification (interaction) to determine whether associations between implementation strength and child mortality differ by socio-economic status, distance to hospital, or geographic zone. Including such checks could reveal important subgroup variations and strengthen interpretation of results.

Discussion

1. The explanation for the lack of association with neonatal and infant mortality is attributed mainly to sample size (lines 432–434). While this is plausible, other reasons (e.g., quality of intrapartum care, referral system issues) could be briefly discussed for completeness.

2. The authors suggest that inclusion of hospitals might have revealed stronger effects, but they do not explore other structural or process factors (e.g., quality of emergency obstetric care) that might explain the null results.

3. They acknowledge PCA limitations (lines 518–522), but the rationale for using a hybrid approach (weighted-additive + PCA) could be better justified in the discussion rather than only in the methods.

4. The authors note that the data are over 10 years old (lines 542–544) but argue that the interventions are still relevant. This argument is valid but could be strengthened by referencing current trends in PHC in Tanzania or citing updated DHS/MNCH program data.

Reviewer #2: The article is written in a tangable manner. However, the study was run from 2011-2015 data which is beyond 10 years. If the research is done more than 5 years ago, it could have less value to the community and programmers to timely recomendation and intervention.

**********

what does this mean? ). If published, this will include your full peer review and any attached files.

**Do you want your identity to be public for this peer review?** If you choose “no”, your identity will remain anonymous but your review may still be made public.

For information about this choice, including consent withdrawal, please see our Privacy Policy

Reviewer #1: **Yes: ** Ameer Muhammad

Reviewer #2: No

**********

[NOTE: If reviewer comments were submitted as an attachment file, they will be attached to this email and accessible via the submission site. Please log into your account, locate the manuscript record, and check for the action link "View Attachments". If this link does not appear, there are no attachment files.]

While revising your submission, please upload your figure files to the Preflight Analysis and Conversion Engine (PACE) digital diagnostic tool, https://pacev2.apexcovantage.com/. PACE helps ensure that figures meet PLOS requirements. To use PACE, you must first register as a user. Registration is free. Then, login and navigate to the UPLOAD tab, where you will find detailed instructions on how to use the tool. If you encounter any issues or have any questions when using PACE, please email PLOS at figures@plos.org. Please note that Supporting Information files do not need this step.

# Author comment: Author response to Decision Letter 1

**DOI:** 10.1371/journal.pgph.0005346.r002

28 Sep 2025

Attachment Submitted filename: Response to Reviewers (9 28).odt

# Editor report: Decision Letter 1

**DOI:** 10.1371/journal.pgph.0005346.r003

**Authors:** Shiyam Sunder

2 Oct 2025

Measuring the strength of maternal, newborn and child health care implementation and its association with childhood mortality risk in three rural districts of Tanzania.

PGPH-D-25-01695R1

Dear Dr. Baynes,

We are pleased to inform you that your manuscript 'Measuring the strength of maternal, newborn and child health care implementation and its association with childhood mortality risk in three rural districts of Tanzania.' has been provisionally accepted for publication in PLOS Global Public Health.

Before your manuscript can be formally accepted you will need to complete some formatting changes, which you will receive in a follow up email. A member of our team will be in touch with a set of requests.

Please note that your manuscript will not be scheduled for publication until you have made the required changes, so a swift response is appreciated.

IMPORTANT: The editorial review process is now complete. PLOS will only permit corrections to spelling, formatting or significant scientific errors from this point onwards. Requests for major changes, or any which affect the scientific understanding of your work, will cause delays to the publication date of your manuscript.

If your institution or institutions have a press office, please notify them about your upcoming paper to help maximize its impact. If they'll be preparing press materials, please inform our press team as soon as possible -- no later than 48 hours after receiving the formal acceptance. Your manuscript will remain under strict press embargo until 2 pm Eastern Time on the date of publication. For more information, please contact globalpubhealth@plos.org.

Thank you again for supporting Open Access publishing; we are looking forward to publishing your work in PLOS Global Public Health.

Best regards,

Shiyam Sunder, MBBS, MSc epidemiology, PhD

Academic Editor

PLOS Global Public Health

***********************************************************

Reviewer Comments (if any, and for reference):
